# Integrative Analyses Reveal the Physiological and Molecular Role of Prohexadione Calcium in Regulating Salt Tolerance in Rice

**DOI:** 10.3390/ijms25169124

**Published:** 2024-08-22

**Authors:** Rui Deng, Yao Li, Nai-Jie Feng, Dian-Feng Zheng, You-Wei Du, Aaqil Khan, Ying-Bin Xue, Jian-Qin Zhang, Ya-Nan Feng

**Affiliations:** 1 College of Coastal Agriculture Sciences, Guangdong Ocean University, Zhanjiang 524088, China; dengrzxc@163.com (R.D.); sdly1997@163.com (Y.L.);; 2 South China Center of National Saline—Tolerant Rice Technology Innovation Center, Zhanjiang 524088, China; 3 Shenzhen Research Institute, Guangdong Ocean University, Shenzhen 518108, China

**Keywords:** prohexadione calcium, salt stress, rice, transcriptome, metabolome, photosynthesis

## Abstract

Salinity stress severely restricts rice growth. Prohexadione calcium (Pro-Ca) modulation can effectively alleviate salt stress in rice. In this study, we explored the effects of Pro-Ca on enhancing salt tolerance in two rice varieties, *IR29* and *HD96-1*. The results revealed that Pro-Ca markedly enhanced root and shoot morphological traits and improved plant biomass under salt stress. Chlorophyll a and b content were significantly increased, which improved photosynthetic capacity. Transcriptomic and metabolomic data showed that Pro-Ca significantly up-regulated the expression of genes involved in E3 ubiquitin ligases in *IR29* and *HD96-1* by 2.5-fold and 3-fold, respectively, thereby maintaining Na^+^ and K^+^ homeostasis by reducing Na^+^. Moreover, Pro-Ca treatment significantly down-regulated the expression of *Lhcb1*, *Lhcb2*, *Lhcb3*, *Lhcb5*, and *Lhcb6* in IR29 under salt stress, which led to an increase in photosynthetic efficiency. Furthermore, salt stress + Pro-Ca significantly increased the *A-AAR* of *IR29* and *HD96-1* by 2.9-fold and 2.5-fold, respectively, and inhibited endogenous cytokinin synthesis and signal transduction, which promoted root growth. The current findings suggested that Pro-Ca effectively alleviated the harmful effects of salt stress on rice by maintaining abscisic acid content and by promoting oxylipin synthesis. This study provides a molecular basis for Pro-Ca to alleviate salt stress in rice.

## 1. Introduction

Salinity stress is a profound environmental issue globally that has noxious effects on agricultural production, resulting in low grain yields and quality [1,2]. Presently about 20% of the world’s cropland and 33% of irrigated lands are saline [3]. Saline soils are variously classified across the world, accounting for 37.42%, 33.43%, 15.39%, 8.43%, and 5.32% in Oceania, Asia, America, Africa, and Europe, respectively [2]. Worldwide, the area of saline lands is increasing due to global warming and the mismanagement of irrigation and fertilization [4].

Rice (*Oryza sativa* L.) is a staple food crop for more than half of the world’s population [1,5]. Rice crops are highly sensitive to salt stress, and salt stress produces ionic hazards and osmotic stresses [6]. Salt stress promotes osmotic stress, which inhibits the ability of rice to absorb water and other nutrients [7]. Ionic toxicity is mainly due to the accumulation of excessive Na^+^ and Cl^−^ in the late stage of salt stress, which eventually reaches toxic levels [8]. It further disrupts the osmotic pressure and water potential of rice cell membranes, which ultimately destroys the integrity of cell membranes [9,10]. These primary stresses lead to oxidative stress and may result in a series of secondary stresses that impact rice growth and development [11]. The entry of a high concentration of salt into rice plants will eventually increase the toxicity level of adult leaves and cause leaf senescence, which is the most direct consequence of cell death in plants and is characterized by synergetic changes at the physiological and molecular level, such as chlorophyll degradation, biosynthesis obstruction, membrane integrity damage, ROS and MDA accumulation, etc. [12]. In order to deal with salt stress, rice plants have the ability to develop a complex adaptive mechanism through long-term evolution [13,14]: maintaining the Na^+^-K^+^ ion balance via channel proteins and transporters [15]; and the improvement of plant resistance to oxidative stress by increasing antioxidant enzyme activity and increasing antioxidant content [5]. Among these, the maintenance of ionic homeostasis occupies an important position in plant resistance to salt stress. Na^+^/H^+^ reverse transporter proteins (*NHXs*) are very important players in this process. Most *NHXs* are located in the plasma and vesicular membranes and are essential for maintaining a low intracellular Na^+^ concentration by releasing Na^+^ into the extracellular fraction and vesicles [16].

Previous studies have found that exogenous plant growth regulators can improve the resistance of rice to salt stress [17]. Prohexadione calcium (Pro-Ca) is a novel plant growth regulator and is a naturally derived cyclohexanecarboxylic acid, which can enhance the crop grain yield and improve salt tolerance without residual toxicity to crops [18]. Previous evidence has shown that an appropriate concentration of Pro-Ca can alleviate salt stress and might maintain cellular osmotic potential by regulating osmotic adjustment molecules, enhancing the activity of antioxidant enzymes, reducing the Malondialdehyde (MDA) content, eliminating reactive oxygen species (ROS), protecting the cell membrane structure, and reducing the degree of membrane peroxidation, thus alleviating the damage caused by salt stress on seedlings and improving the ability of seedlings to resist salt [19]. The foliar application of Pro-Ca significantly increased chlorophyll a (Chla), chlorophyll b (Chlb), chlorophyll a+b (Chla+b), and carotenoid (Car) contents, Fv/Fm and Fv/Fo, and maintained photosystem II (PSII) activity under salt stress [20]. In addition, Pro-Ca increased ascorbic acid (ASA) content and enhanced superoxide dismutase (SOD), peroxidase (POD), catalase (CAT), and ascorbate peroxidase (APX) activities in rice [18].

Photosynthesis is the most important physicochemical process for energy production in higher plants, and it is very sensitive to salt stress [21]. Excessive ion concentrations induce stomatal closure, to reduce water loss through transpiration and decrease intercellular carbon dioxide concentrations, leading to a decrease in the net photosynthetic rate [22,23]. A previous study showed that salt stress inhibited the net photosynthetic rate (Pn), stomatal conductance (Gs), intercellular CO_2_ concentration (Ci), transpiration rate (Tr) and various chlorophylls in chloroplasts [24]. In higher plants, LHC II is one of the photosynthetic pigment–protein complexes comprised of intrinsic transmembrane helices holding chlorophyll a (Chl a) and chlorophyll b (Chl b) that are used for harvesting light energy and energy transmission to PSII and PSI [25]. Lhc has been considered a standard plant gene which encodes the chlorophyll a/b binding protein [26]. The light-harvesting pigment–protein complex (LHCH II) of photosystem II is mainly composed of six pigment–protein complexes: LHCB1, LHCB2, LHCB3, LHCB4, LHCB5, and LHCB6 [27]. These six pigment–protein complexes consist of proteins and pigments formed by the six genotypes encoded by *Lhcb1*, *Lhcb2*, *Lhcb3*, *Lhcb4*, *Lhcb5*, and *Lhcb6*, respectively [28]. LHCB1, LHCB2, and LHCB3 are collectively referred to as the main body LHC II, and LHCB4, LHCB5, and LHCB6 are collectively referred to as the trace LHC II. *Lhc* gene expression is dominant when light collection restricts plant growth under low light. *Lhc* gene expression is down-regulated under high light conditions, because of the lower need for effective light trapping under high light conditions [26].

Previous studies investigated the physiological and biochemical mechanism of rice tolerance to salt stress via the foliar application of Pro-Ca. However, the molecular mechanism of salt tolerance and the relationship between the transcriptome and metabolome have not yet been clarified. In this study, we explored the role of the foliar application of Pro-Ca by evaluating the physiological and molecular mechanisms of rice under salt stress. In the current study, an integrated analysis of the metabolome (LC-MS/MS) and transcriptome was used to investigate the molecular mechanisms of salt tolerance in the rice varieties *IR29* (salt-sensitive rice) and *HD96-1* (salt-tolerant rice). Morpho-physiological traits were analyzed to identify molecular mechanisms. Differences in metabolites and genes were compared before and after Pro-Ca treatments under salt stress. This work will further refine our understanding of the physiological and molecular mechanisms of prohexadione calcium, to effectively improve salt tolerance in rice.

## 2. Result

### 2.1. Effect of Pro-Ca on the Phenotype of Rice Seedlings under Salt Stress

**Figure 1:**
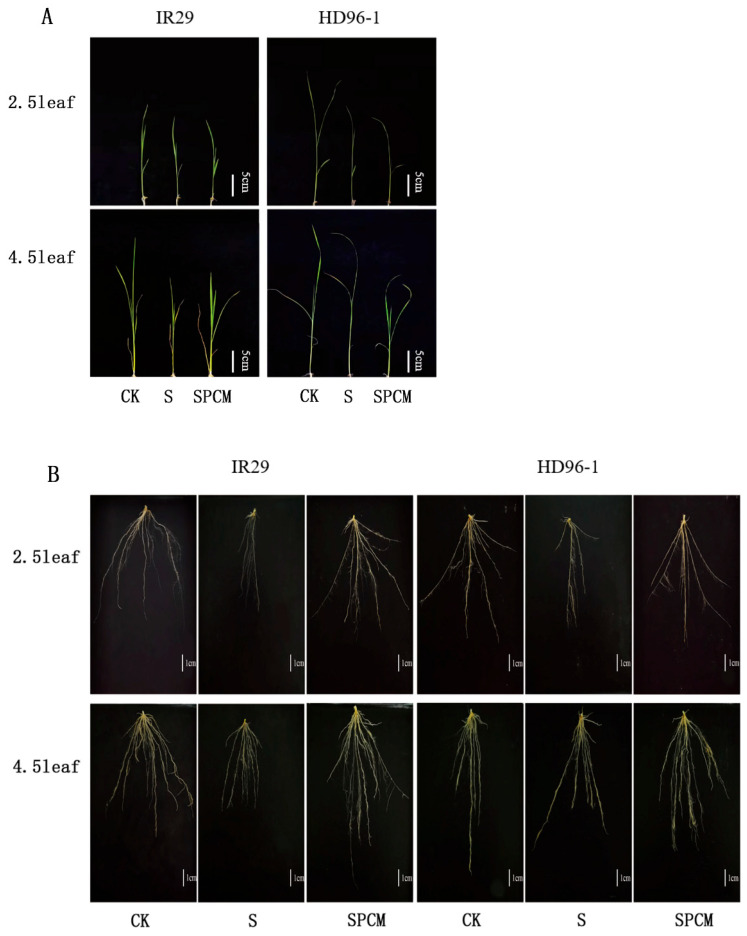
Effect of Pro-Ca treatment on phenotypes of above-ground parts (**A**) and underground parts (**B**) in IR29 and HD96-1 varieties under salt stress at 2.5 and 4.5 leaf stages. CK: 0 mM NaCl + 0 mg·L^−1^Pro-Ca, S: 50 mM NaCl + 0 mg·L^−1^Pro-Ca, SPCM: 50 mM NaCl + 100 mg·L^−1^Pro-Ca.

Salt stress significantly inhibited the root growth, plant height, stem basal width, and leaf area of both varieties in comparison to control (Figure 1).

**Figure 2:**
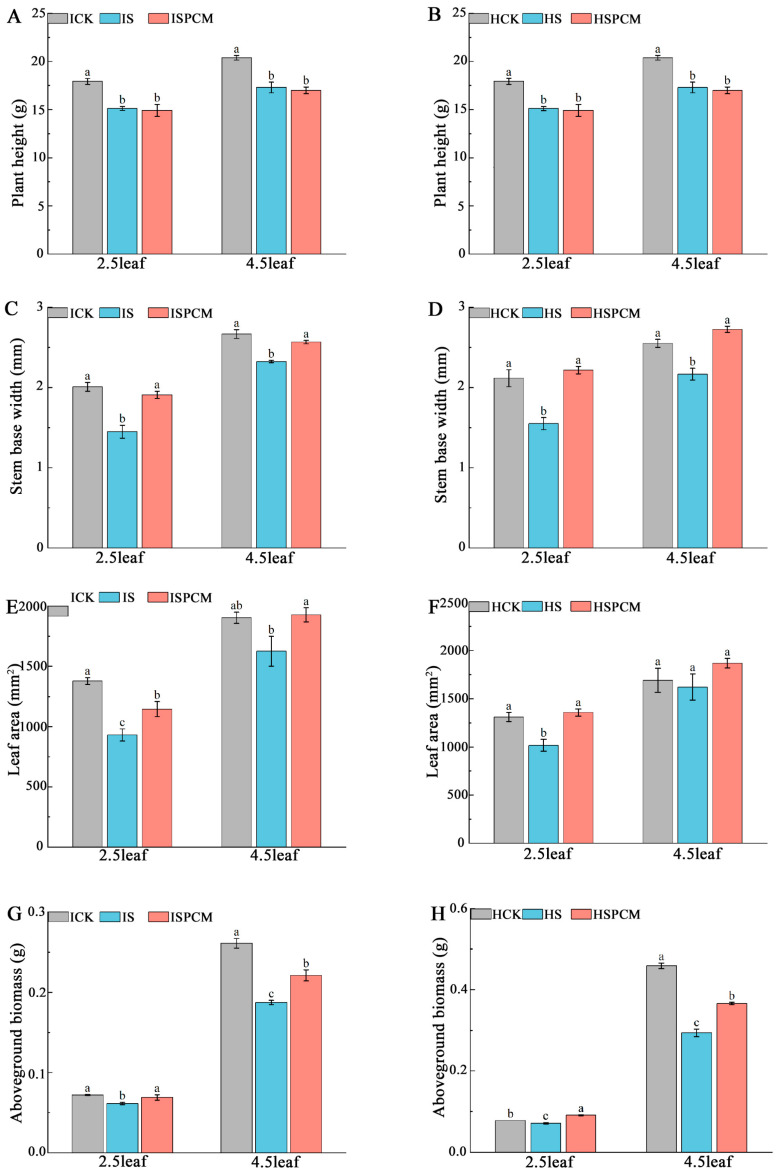
Effect of Pro-Ca treatment on phenotypes of above-ground parts of IR29 and HD96-1 varieties at 2.5 and 4.5 leaf stages under salt stress. Plant height in IR29 (**A**) and HD96-1 (**B**); stem base width in IR29 (**C**) and HD96-1 (**D**); leaf area in IR29 (**E**) and HD96-1 (**F**); and above-ground biomass in IR29 (**G**) and HD96-1 (**H**). CK: 0 mM NaCl + 0 mg·L^−1^Pro-Ca, S: 50 mM NaCl + 0 mg·L^−1^Pro-Ca, SPCM: 50 mM NaCl + 100 mg·L^−1^Pro-Ca. Values are the mean ± SE of three replicate samples. Different letters in the data column indicate significant differences (*p* < 0.05) according to Duncan’s test.

Salt stress treatments significantly reduced the stem basal width, leaf area, and above-ground dry matter (Figure 2). At the 2.5 leaf stage, the S treatment significantly decreased plant height by 15.66% and 5.17%, stem basal width by 27.79% and 26.78%, leaf area by 32.48% and 22.48%, and above-ground dry matter by 15.28% and 8.97%, respectively, for *IR29* and *HD96-1*, compared with CK. (Figure 2). The stem basal width of *IR29* decreased from 2.0083 mm to 1.45 mm, and that of *HD96-1* decreased from 2.12 mm to 1.55 mm in the control. The leaf area of *IR29* decreased from 1380.07 mm^2^ to 931.87 mm^2^, and that of *HD96-1* decreased from 1311.03 mm^2^ to 1016.37 mm^2^. The dry weight of the above-ground portion decreased from 0.072 g to 0.061 g in *IR29* and from 0.078 g to 0.071 g in *HD96-1*.

Compared to the S treatment, the combined treatments of Pro-Ca and salt stress significantly increased stem basal width by 31.59% and 43.03%, leaf area by 23.05% and 33.6%, and above-ground dry matter by 13.11% and 28.17%, respectively, for *IR29* and *HD96-1*. The stem basal width of *IR29* increased significantly from 1.45 mm to 1.908 mm, and that of *HD96-1* increased from 1.55 mm to 2.217 mm. The leaf area of *IR29* increased from 931.867 mm^2^ to 1146.7 mm^2^, and that of *HD96-1* increased significantly from 1016.367 mm^2^ to 1357.833 mm^2^. The dry weight of the above-ground portion increased from 0.061 g to 0.069 g in *IR29* and from 0.071 g to 0.091 g in *HD96-1*. At the 2.5 leaf stage, compared with CK, the combined treatments of Pro-Ca and salt stress decreased the plant height, stem basal width, leaf area, and above-ground dry weight of IR29 by 16.82%, 4.98%, 16.91%, and 4.17%, respectively. The combined treatments of Pro-Ca and salt stress decreased the plant height of *HD96-1* by 9.71%. Compared with CK, the combined treatments of Pro-Ca and salt stress increased the stem base width, leaf area, and above-ground dry weight of *HD96-1* by 4.72%, 3.57%, and 16.67%, respectively. Similarly, at the 4.5 leaf stage, the S treatment significantly decreased the stem basal width by 12.82% and 15.02% and above-ground dry matter by 28.35% and 35.95%, respectively, for *IR29* and *HD96-1*, compared with CK. The S treatment slightly decreased leaf area and plant height in *IR29* and *HD96-1* (Figure 2). Stem basal width decreased from 2.67 mm to 2.33 mm in *IR29* and from 2.55 mm to 2.17 mm in *HD96-1*. Above-ground dry weight decreased from 0.261 g to 0.187 g in IR29 and from 0.459 g to 0.294 g in *HD96-1*. Compared to the S treatment, the combined treatments of Pro-Ca and salt stress significantly increased stem basal width by 10.41% and 25.75%, leaf area by 18.6% and 15.19%, and above-ground dry matter by 18.18% and 24.49%, respectively, for *IR29* and *HD96-1*, compared with salt stress treatments. Stem basal width increased from 2.325 mm to 2.567 mm in IR29 and from 2.167 mm to 2.725 mm in *HD96-1*. Above-ground partial dry weight increased from 0.187 mm to 0.221 mm in *IR29* and from 0.294 mm to 0.366 mm in *HD96-1*. At the 4.5 leaf stage, compared with CK, the combined treatments of Pro-Ca and salt stress decreased the plant height, stem basal width and above-ground dry weight of *IR29* by 16.60%, 3.75% and 15.33%, respectively, and slightly increased the leaf area of *IR29* by 1.26%. Compared with the CK treatment at the 4.5 leaf stage, the combined treatments of Pro-Ca and salt stress significantly decreased the plant height and above-ground dry weight of *HD96-1* by 33.56% and 20.26%, respectively, and increased the stem base width and leaf area of *HD96-1* by 6.86% and 10.32%, respectively.

**Figure 3:**
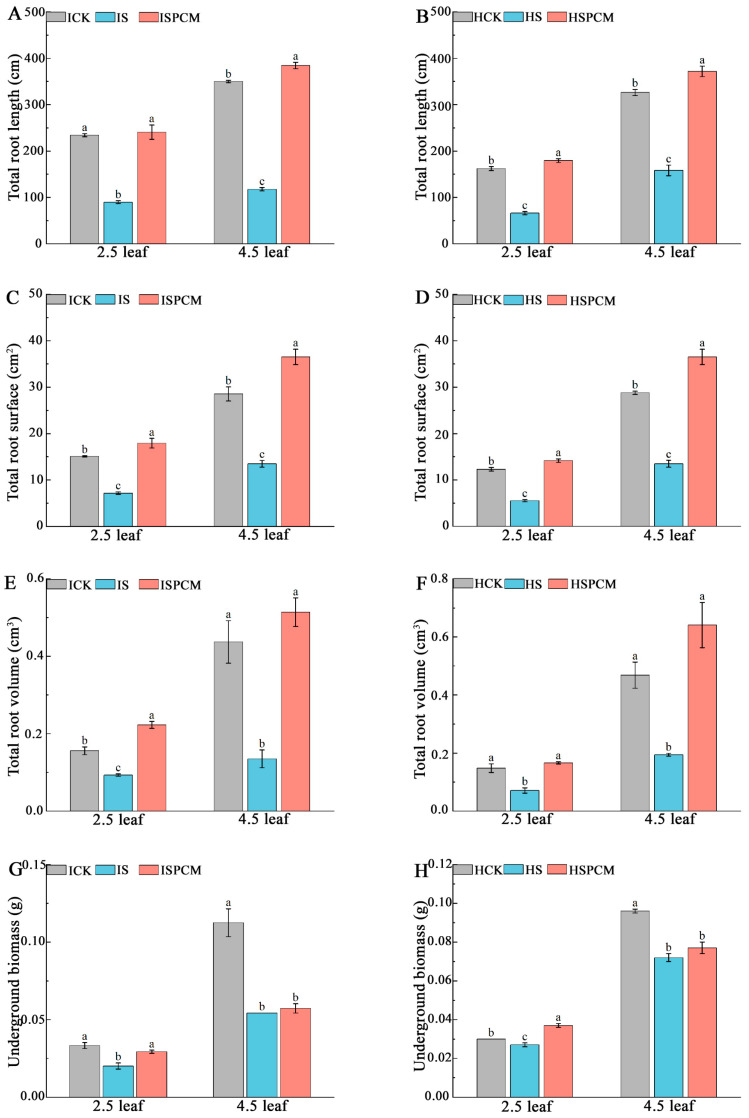
Effect of Pro-Ca on phenotypes of underground parts of IR29 and HD96-1 varieties under salt stress at 2.5 and 4.5 leaf stages. Total root length in IR29 (**A**) and HD96-1 (**B**); total root surface area in IR29 (**C**) and HD96-1 (**D**); total root volume in IR29 (**E**) and HD96-1 (**F**); and underground biomass in IR29 (**G**) and HD96-1 (**H**). CK: 0 mM NaCl + 0 mg·L^−1^Pro-Ca, S: 50 mM NaCl + 0 mg·L^−1^Pro-Ca, SPCM: 50 mM NaCl + 100 mg·L^−1^Pro-Ca. Values are the mean ± SE of three replicate samples. Different letters in the data column indicate significant differences (*p* < 0.05) according to Duncan’s test.

The salt stress treatment significantly inhibited the root growth of both varieties of rice compared with the CK treatment. At the 2.5 leaf stage, the S treatment significantly decreased total root length by 61.71% and 59.23%, total root area by 52.58% and 55.08%, total root volume by 40.38% and 52.03%, and underground dry matter by 39.39% and 10%, respectively, for *IR29* and *HD96-1* under salt stress, compared with CK. Compared to salt stress treatment, the combined treatments of Pro-Ca and salt stress significantly increased total root length by 168.62% and 198.72%, total root area by 150.47% and 156.72%, total root volume by 139.78% and 133.80%, and underground dry weight by 45% and 37.04%, respectively, for *IR29* and *HD96-1* under salt stress (Figure 3). Similarly, at the 4.5 leaf stage, the S treatment significantly decreased total root length by 66.42% and 51.51%, total root area by 67.83% and 53.17%, total root volume by 69.11% and 58.55%, and underground dry weight by 52.21% and 25%, respectively, for *IR29* and *HD96-1*, compared with the CK treatments (Figure 3). At the 4.5 leaf stage, compared to the S treatment, the combined treatments of Pro-Ca and salt stress significantly increased total root length by 226.97% and 135.06%, total root area by 252.03% and 171.01%, total root volume by 280.74% and 230.41%, and underground dry weight by 5.56% and 6.94% (the underground dry weight showing no significant change), respectively, for *IR29* and *HD96-1*, compared with salt stress treatments (Figure 3). At the 2.5 leaf stage, the dry weight of the underground portion of IR29 significantly decreased from 0.033 g to 0.02 g under salt stress and increased to 0.029 g after Pro-Ca treatment. The dry weight of the underground portion of *HD96-1* decreased from 0.03 g to 0.027 g under salt stress and increased to 0.037 g after Pro-Ca treatment. At the 4.5 leaf stage, the dry weight of the underground portion of *IR29* decreased from 0.113 g to 0.054 g under salt stress and increased to 0.057 g after Pro-Ca treatment. The dry weight of the underground part of *HD96-1* decreased from 0.096 g to 0.072 g under salt stress and increased to 0.077 g after Pro-Ca treatment. At the 2.5 leaf stage, compared to the S treatment, the combined treatments of Pro-Ca and salt stress increased the total root length, total root surface area, and total root volume of *IR29* by 2.85%, 18.78%, and 42.95%, respectively, and significantly decreased the underground dry weight of IR29 by 12.12%. Compared with the CK treatment, the combined treatments of Pro-Ca and salt stress significantly increased the total root length, total root surface area, total root volume, and underground dry weight of *HD96-1* by 10.67%, 15.02%, 12.16%, and 18.92%, respectively. At the 4.5 leaf stage, compared with CK, the combined treatments of Pro-Ca and salt stress increased the total root length, total root surface area, and total root volume of *IR29* and *HD96-1* by 9.8% and 13.98%, 28.01% and 26.92%, and 17.62% and 36.97%, respectively. Compared with CK, the combined treatments of Pro-Ca and salt stress decreased the underground dry weight by 49.56% and 19.79%, respectively, for *IR29* and *HD96-1*.

### 2.2. Effect of Pro-Ca on the Homeostasis of K^+^ and Na^+^ in Rice under Salt Stress

Compared to the CK treatment, the salt stress treatment significantly increased leaf Na^+^ content by 1283.13% and 1121.18%, root Na^+^ content by 399.37% and 301.88%, leaf Na^+^/K^+^ by 1300% and 1300%, and root Na^+^/K^+^ by 460% and 360%, and decreased leaf K^+^ content by 2.77% and 15.31% and root K^+^ content by 14.33% and 15.16%, respectively, for *IR29* and *HD96-1* under salt stress (Table 1).

Compared to the S treatment, the combined treatments of Pro-Ca and salt stress significantly decreased leaf Na^+^ content by 86.32% and 91.33%, root Na^+^ content by 81.36% and 71.38%, leaf Na^+^/K^+^ by 87.50% and 92.86%, and root Na^+^/K^+^ by 85.71% and 76.09%, and increased leaf K^+^ content by 7.42% and 17.24% and root K^+^ content by 31.41% and 21.49%, respectively, for *IR29* and *HD96-1* under salt stress (Table 1).

The research data indicated that the rice accumulated a large amount of Na^+^ in both rice varieties under salt stress, which inhibited the uptake of other ions and disrupted the Na^+^/K^+^ balance. Pro-Ca and salt stress combination treatments significantly reduced the effects of salt stress on Na^+^ accumulation in the above-ground parts and roots and increased K^+^ content in the above-ground parts and roots of rice under salt stress, maintaining ionic homeostasis and improving salt tolerance.

### 2.3. Effects of Pro-Ca on Photosynthesis- and Fluorescence-Related Parameters of Rice Leaves under Salt Stress

Salt stress significantly reduced the photosynthetic pigment content of both varieties, while Pro-Ca significantly increased the photosynthetic pigment content of both varieties.

At the 2.5 leaf stage, there were significantly decreasing trends of 22.35% in Chl a content, 26.42% in Chl b content, 23.42% in Chl a+b, and 20.92% in carotenoid content for *IR29* under salt stress, compared with CK. At the same time, the above parameters for *HD96-1* slightly decreased (Table 2). Compared to the S treatment, the combined treatments of Pro-Ca and salt stress increased Chl a by 4.39% and 5.90%, Chl b by 3.83% and 7.43%, and Chl a+b by 4.25% and 6.27%, respectively, for *IR29* and *HD96-1* under salt stress (Table 2). At the 4.5 leaf stage, there were significantly decreasing trends of 18.39% and 19.70% in Chl a, 22.68% and 39.87% in Chl b, 19.50% and 26.17% in Chl a+b, and 18.63% and 9.00% in carotenoid content, respectively, for *IR29* and *HD96-1* under salt stress, compared with CK treatments (Table 2). Compared to the S treatment, the combined treatments of Pro-Ca and salt stress increased Chl a by 23.32% and 12.53%, Chl b by 36.85% and 35.95%, and Chl a+b by 26.66% and 17.74%, respectively, for *IR29* and *HD96-1* under salt stress. And carotenoids were significantly increased by 23.75% in *IR29* and slightly increased by 3.07% in *HD96-1* (Table 2).

Rice under salt stress significantly reduced the value of its gas exchange parameters compared to CK. Compared with the salt stress treatment, the foliar application of Pro-Ca significantly alleviated the inhibition of photosynthesis by salt stress, as seen by the increase in P_n_, G_s_, C_i_, and Tr in rice.

**Figure 4:**
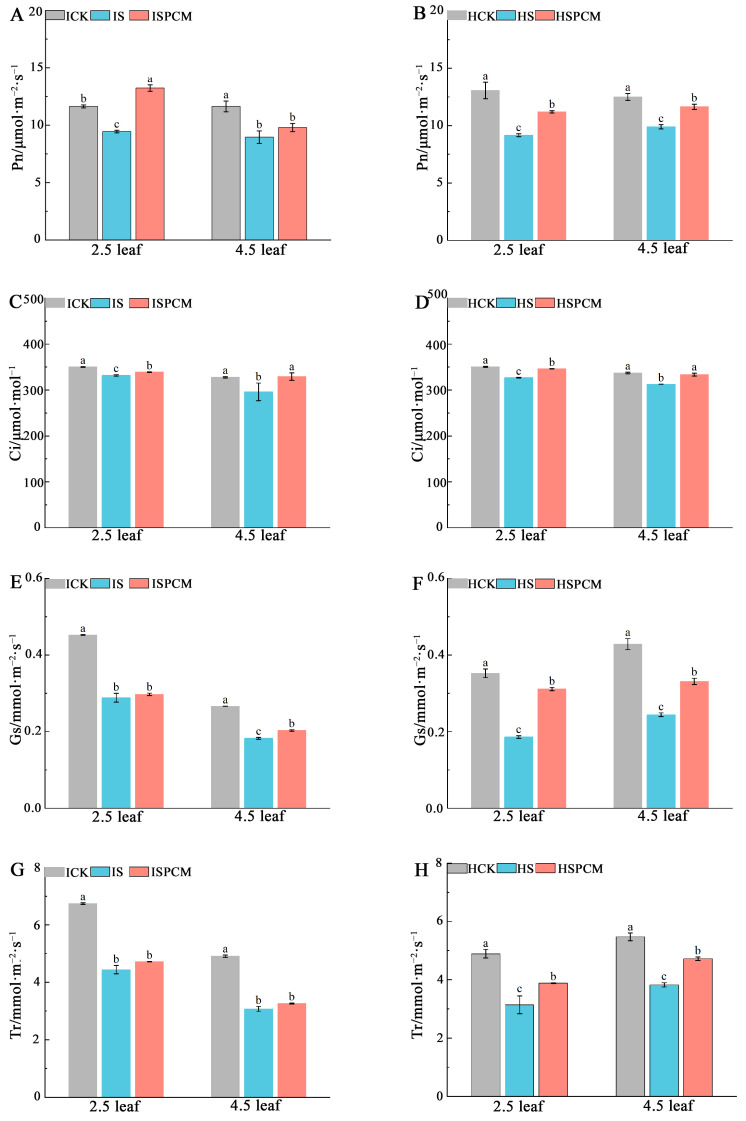
The effect of Pro-Ca on rice seedlings gas exchange parameters under salt stress. P_n_ in IR29 (**A**) and HD96-1 (**B**); C_i_ in IR29 (**C**) and HD96-1 (**D**); G_s_ in IR29 (**E**) and HD96-1 (**F**); and T_r_ in IR29 (**G**) and HD96-1 (**H**). CK: 0 mM NaCl + 0 mg·L^−1^Pro-Ca, S: 50 mM NaCl + 0 mg·L^−1^Pro-Ca, SPCM: 50 mM NaCl + 100 mg·L^−1^Pro-Ca. Values are the mean ± SE of three replicate samples. Different letters in the data column indicate significant differences (*p* < 0.05).

At the 2.5 leaf stage, the S treatment decreased P_n_ by 18.83% and 29.87%, G_s_ by 36.26% and 47.11%, C_i_ by 5.23% and 6.75%, and T_r_ by 34.12% and 35.86%, respectively for *IR29* and *HD96-1*, compared with CK (Figure 4). Compared to salt stress treatment, the combined treatments of Pro-Ca and salt stress increased P_n_ by 40.13% and 22.23%, G_s_ by 3.01% and 66.91%, Ci by 2.11% and 5.91%, and Tr by 6.23% and 23.49%, respectively, for *IR29* and *HD96-1* under salt stress. Similarly, for salt stress treatments at the 4.5 leaf stage, there were significantly decreasing trends of 22.98% and 20.83% in P_n_, 31.5% and 42.96% in G_s_, 9.66% and 7.31% in C_i_, and 37.5% and 30.12% in T_r_, respectively, for *IR29* and *HD96-1*, compared with CK treatments (Figure 4). Compared to salt stress treatment, the combined treatments of Pro-Ca and salt stress increased P_n_ by 9.26% and 17.75%, G_s_ by 11.31% and 35.47%, and Ci by 11.26% and 6.72%, respectively, for *IR29* and *HD96-1*. Meanwhile, Tr was significantly increased by 23.47% in *HD96-1* and slightly increased in *IR29* (Figure 4).

**Figure 5:**
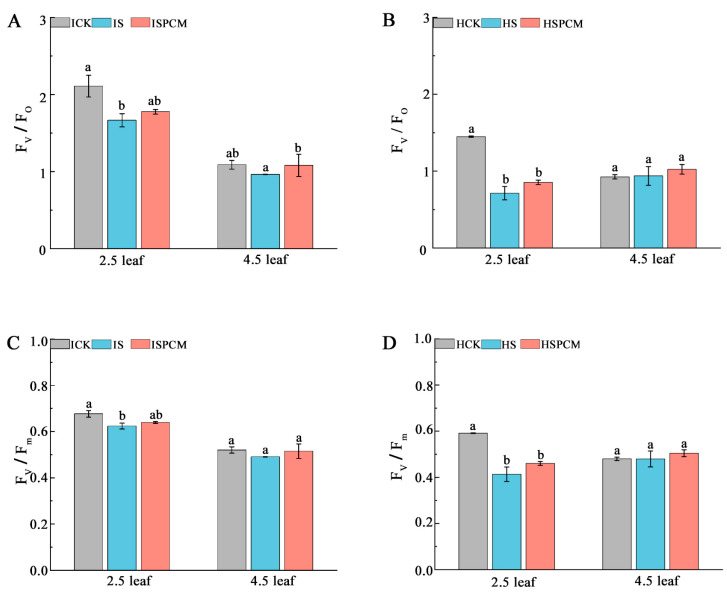
Effect of Pro-Ca on chlorophyll fluorescence parameters of rice seedlings under salt stress. F_v/_F_o_ in IR29 (**A**) and HD96-1 (**B**); and F_v/_F_m_ in IR29 (**C**) and HD96-1 (**D**). Mean ± SE of three replicates. Different letters indicate significant differences (*p* < 0.05). CK: 0 mM NaCl + 0 mg·L^−1^Pro-Ca, S: 50 mM NaCl + 0 mg·L^−1^Pro-Ca, SPCM: 50 mM NaCl + 100 mg·L^−1^Pro-Ca. Values are the mean ± SE of three replicate samples. Different letters in the data column indicate significant differences (*p* < 0.05).

At the 2.5 leaf stage, the S treatment significantly decreased F_v_/F_o_ by 21.05% and 50.76%, respectively, for *IR29* and *HD96-1* under salt stress, compared with CK (Figure 2A,B). Compared with the salt stress treatments, the F_v_/F_o_ of both rice Pro-Ca and salt stress combination treatments increased to different degrees, with significant increases of 6.61% and 19.67% for *IR29* and *HD96-1*, respectively, at the 2.5-leaf stage (Figure 5A,B); at the 4.5-leaf stage, significant increases of 12.14% for *IR29* and 9.10% for *HD96-1* were observed, but the differences did not reach the significant level (Figure 5A,B). The F_v_/F_m_ of the rice seedlings decreased after salt stress; the salt stress treatments of *IR29* and *HD96-1* significantly decreased F_v_/F_m_ by 7.83% and 30.14% at the 2.5-leaf stage compared with the CK treatment, whereas the difference did not reach a significant level at the 4.5-leaf stage (Figure 5C,D). After the combination treatment of Pro-Ca and salt stress, the F_v_/F_m_ compared with the salt treatment all showed different degrees of increase; the Pro-Ca and salt stress combination treatments of *IR29* and *HD96-1* significantly increased F_v_/F_m_ by 2.51% and 11.38% at the 2.5-leaf stage compared with the salt treatment, whereas the difference did not reach a significant level at the 4.5-leaf stage (Figure 5C,D).

### 2.4. Analysis of Endogenous Plant Hormone Content

The content of three cytokinins, meta-Topolin-9-glucoside, Dihydrozeatin-O-glucoside riboside, and 6-Benzyladenine, was significantly decreased in *IR29* by the combination treatment of Pro-Ca and salt stress compared to salt stress. The content of two cytokinins, cis-Zeatin and Kinetin-9-glucoside, in *HD96-1* also showed a decreasing trend (Table 3).

### 2.5. Effect of Pro-Ca Treatment on Rice Transcriptome under Salt Stress

#### 2.5.1. Reliability Analysis

**Figure 6:**
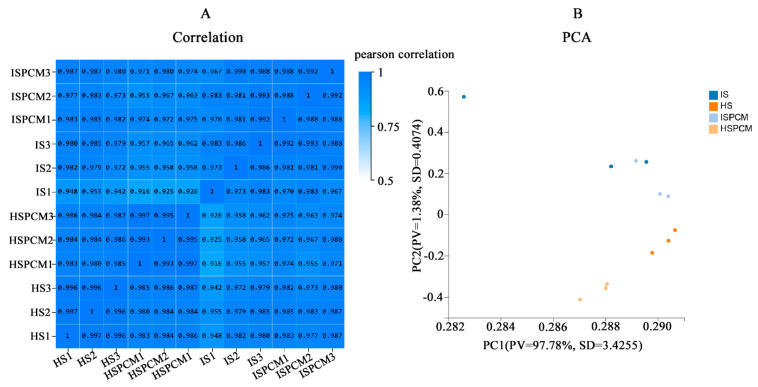
Overall analysis of the transcriptome. (**A**) Pearson correlation coefficients between the samples. (**B**) Principal component analysis of the samples. S: salt stress treatment (50 mM NaCl + 0 mg·L^−1^Pro-Ca); SPCM: Pro-Ca and salt stress combination treatment (50 mM NaCl + 100 mg·L^−1^Pro-Ca).

To reflect the correlation of gene expression between samples, Pearson correlation coefficients (PCCs) were calculated for all gene expressions between each of the two samples, and these coefficients were reflected in the form of heatmaps (Figure 6A). For all treatments, we could observe a high correlation between biological replicates, which indicates the reliability of the biological replicates in this study. Principal component analysis (PCA) showed that PC1 captured most of the variance in the data (96.63%) and PC2 captured a lesser variance in the data (1.78%) (Figure 6B), which varied significantly between treatments and also varied between species.

RT-qPCR was used to detect the expression levels of seven identified transcriptome (RNA-ref) differential genes to verify the RNA-ref data. The results of the RT-qPCR are positively correlated with those of the RNA-ref (Table S1), demonstrating that the RNA sequencing data were reliable.

#### 2.5.2. Comparative Analysis of Differentially Expressed Genes under Salt Stress

**Figure 7:**
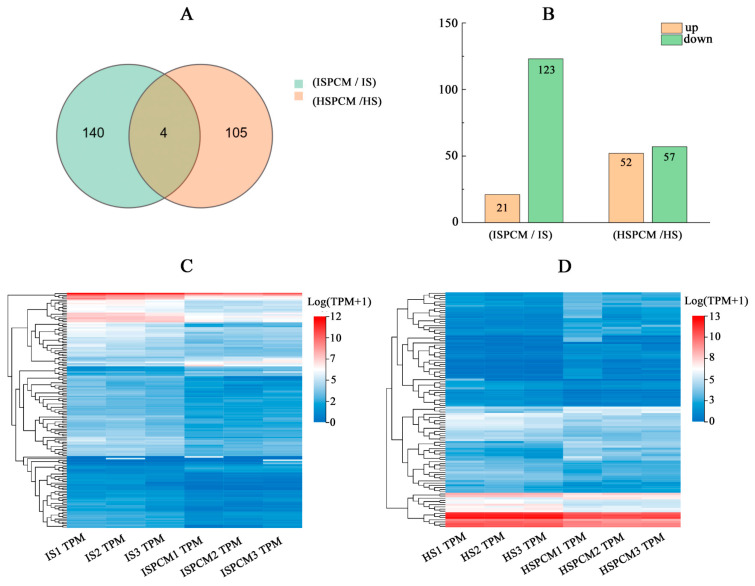
Venn diagram analysis of differential genes in IR29 and HD96-1 (**A**) seedlings under different treatments, and analysis of differentially up-regulated and down-regulated genes (**B**) in IR29 and HD96-1 seedlings under different treatments. Cluster analysis of all DEGs in IR29 (**C**) and HD96-1 (**D**) seedlings under different treatments. S: salt stress treatment (50 mM NaCl + 0 mg·L^−1^Pro-Ca); SPCM: Pro-Ca and salt stress combination treatment (50 mM NaCl + 100 mg·L^−1^Pro-Ca). Differential genes were determined using |Log2FC|≥ 1 and q < 0.05.

The authors selected |Log2FC| ≥ 1 and q < 0.05 significantly differentially expressed genes and performed a comparative Venn diagram analysis of significantly differentially expressed genes in different rice varieties before and after Pro-Ca treatment under salt stress. The results showed that there were many unique salt-responsive genes in *IR29* and *HD96-1*. Compared with the salt stress treatment, there were 140 and 105 unique differentially expressed genes in the combination of Pro-Ca and salt stress treatments in *IR29* and *HD96-1*, respectively, and there were four shared differentially expressed genes (Figure 7A and Table S2). In the ISPCM/IS and HSPCM/HS groups, the expression of 21 and 52 DEGs was significantly up-regulated, and the expression of 123 and 57 DEGs was significantly down-regulated, respectively (Figure 7B). A cluster analysis showed that Pro-Ca significantly altered the differential expression of the two rice genes (Figure 7C,D).

**Figure 8:**
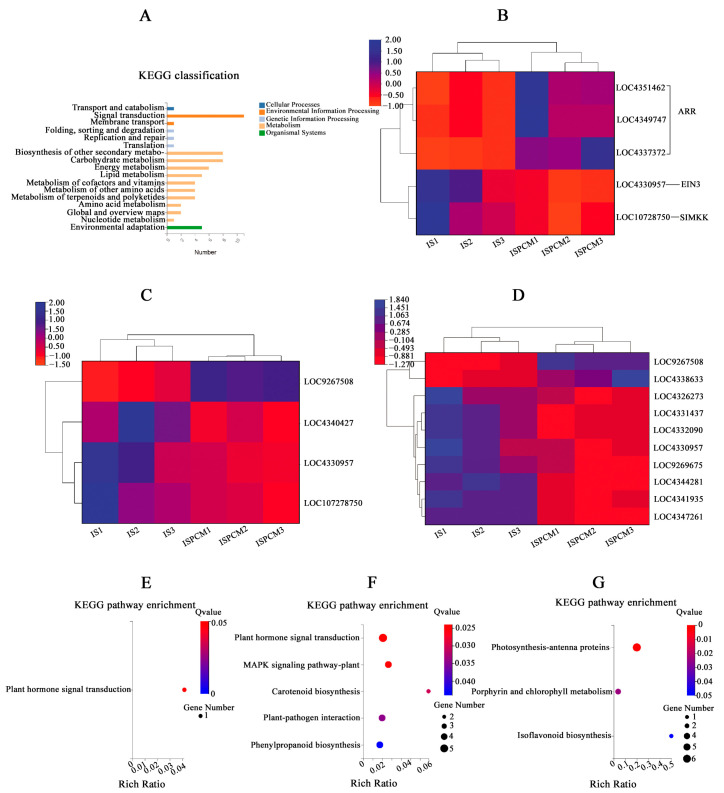
KEGG annotation map of all significantly differentially expressed genes in ISPCM/IS vs. HSPCM/HS (**A**). Expression profiles of DEGs related to plant hormone signal transduction (**B**), MAPK plant signaling (**C**), and carotenoid biosynthesis, phenylpropane biosynthesis, and plant-pathogen interactions (**D**). Differentially expressed gene-enriched KEGG pathway specific to HSPCM/HS (**G**), ISPCM/IS (**F**), and significant enrichment of the KEGG pathway with differentially expressed genes common to the ISPCM/IS and HSPCM/HS groups (**E**). S: salt stress treatment (50 mM NaCl + 0 mg·L^−1^Pro-Ca); SPCM: Pro-Ca and salt stress combination treatment (50 mM NaCl + 100 mg·L^−1^Pro-Ca).

**Figure 9:**
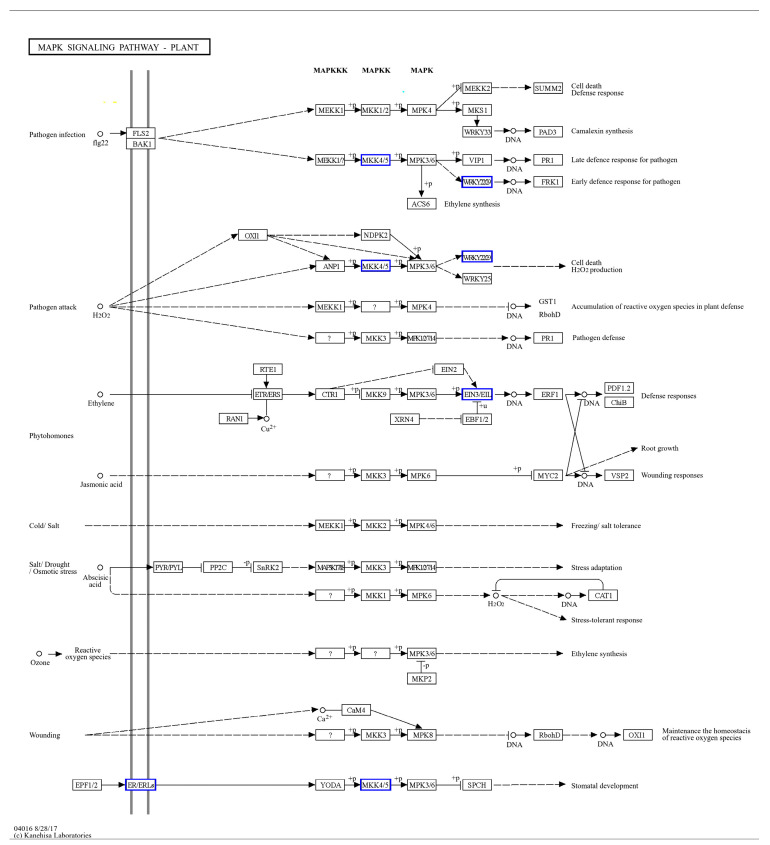
Enrichment of ISPCM/IS differentially expressed genes into the MAPK plant signaling pathway.

All the significantly differentially expressed genes of ISPCM/IS and HSPCM/HS were analyzed by KEGG annotation. The results showed that these genes were mainly enriched in several categories, including “signal transduction”, “secondary metabolite biosynthesis”, “carbohydrate metabolism”, and “energy metabolism” (Figure 8A). Compared with the salt stress treatment, the two rice varieties had four identical differentially expressed genes, including “*LOC4342341*”, “*LOC4343997*”, “*LOC4349747*”, and “*LOC9269432*”, following the combination of Pro-Ca and salt stress treatment. These four genes may be candidates for Pro-Ca to alleviate salt stress in rice. *LOC4343997* is an E3 ubiquitin-protein ligase SIRP1-like gene. The GO function analysis of the four salt-tolerance candidate genes showed that the salt-tolerance candidate genes are significantly related to the “signal transduction” function. A further KEGG pathway enrichment of salt tolerance candidate genes revealed that the “Plant hormone signal transduction” pathway was significantly enriched (Figure 8E). This suggested that the plant hormone signal transduction pathway may be highly correlated with the regulation of Pro-Ca in rice adaptation to salt stress, and we found that important control genes (*LOC4359747*) of *A-ARR* in this pathway showed a significant up-regulation trend (Figure 8B). ISPCM/IS-specific differentially expressed genes were also significantly enriched into four specific pathways: the “plant MAPK signaling pathway”, “carotenoid biosynthesis”, “plant–pathogen interactions”, and “phenylpropanoid biosynthesis”. In addition, “phytohormone signaling” was also significantly enriched (Figure 8F and Table S5), and in addition to the changes in *A-ARR*-related control genes in the plant hormone signal transduction, the down-regulation of *EIN3* control genes related to ethylene synthesis was also found (Figure 8B). The “plant MAPK signaling pathway” was significantly enriched by four differentially expressed genes (Figure 8C): *LOC107278750*, *LOC4330957*, *LOC4340427*, and *LOC9267508*. The differentially expressed genes in the pathway were mainly focused on peptides, endogenous hormone levels, and stomatal development. In terms of polypeptides and hydrogen peroxide, we found that the gene *LOC9267508* related to the transcription factor *WRKY22* was significantly up-regulated under the combined treatment of Pro-Ca and salt stress. The gene *LOC107278750* related to the control of EIN3-5like of the ETH synthesis pathway was significantly down-regulated in the combined Pro-Ca and salt stress treatment compared to the salt treatment. In stomatal development, the gene *OJ1316_E06.24* (*LOC4330957*), which is the relevant control gene for the signaling cascade *MKK4/5* in this process, showed a significant down-regulation in response to Pro-Ca (Figure 9).

KEGG pathways significantly enriched in HSPCM/HS-specific differentially expressed genes included “photosynthesis antenna proteins”, “porphyrin and chlorophyll metabolism”, and “isoflavonoid biosynthesis” (Figure 8G and Table S3). Among them, the expression of *Lhcb1*, *Lhcb2*, *Lhcb3*, *Lhcb5*, and *Lhcb6*, the LHC II-related genes encoding the “photosynthesis antenna proteins” pathway, showed a significant down-regulation, suggesting that the mitigating effect of Pro-Ca on HD96-1 salt stress may be significantly linked to photosynthesis.

### 2.6. Effect of Pro-Ca Treatment on Rice Metabolome under Salt Stress

**Figure 10:**
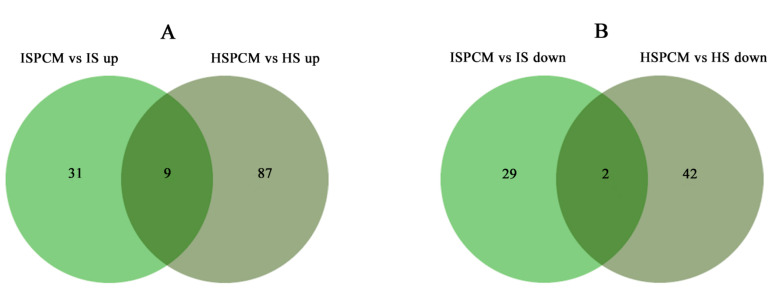
Venn diagram analysis of metabolites with significantly increased (**A**) and decreased (**B**) abundances in IR29 and HD96-1 seedlings under different treatments. S: salt stress treatment (50 mM NaCl + 0 mg·L^−1^Pro-Ca); SPCM: Pro-Ca and salt stress combination treatment (50 mM NaCl + 100 mg·L^−1^Pro-Ca). Differential metabolites were determined using |Log2FC| ≥ 1 and *p* < 0.05.

**Figure 11:**
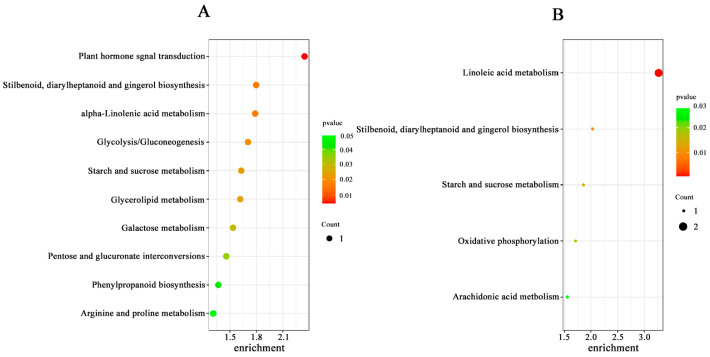
Significant enrichment of the KEGG pathway for differential metabolites in the ISPCM/IS (**A**) and HSPCM/HS (**B**) groups. S: salt stress treatment (50 mM NaCl + 0 mg·L^−1^Pro-Ca); SPCM: Pro-Ca and salt stress combination treatment (50 mM NaCl + 100 mg·L^−1^Pro-Ca).

The mass spectrometry data were collected by LC-MS/MS. In order to find out whether the metabolomic profiles of treated and control plants were different or not, a combination of PCA multivariate statistical analysis and PLS-DA univariate analysis was used to screen for differential metabolites between the groups. VIP ≥ 1, Fold-Change ≥ 1.2 or ≤0.83, and *p*-value < 0.05 were used as the screening criteria for differential metabolites (Table S4). Compared with salt stress, there were significant changes in the levels of 71 metabolites in the combined Pro-Ca and salt stress treatments (ISPCM/IS) for *IR29* and 140 metabolites for *HD96-1* (HSPCM/HS). Compared with the salt stress treatment, the two rice varieties treated with a combination of Pro-Ca and salt stress (ISPCM/IS, HSPCM/HS) had eleven of the same differential metabolites, of which nine were increased and two were reduced (Figure 10A,B). Compared with HSPCM/IS, 31 unique metabolites were increased in the ISPCM/IS group and 29 unique metabolites were decreased in the ISPCM/IS group; 87 and 42 unique differential metabolites were increased and decreased only in HSPCM/HS compared with the ISPCM/IS group, respectively (Figure 10A,B). The 71 differential metabolites of *IR29* were significantly enriched in plant hormone signal transduction, alpha-linolenic acid metabolism, galactose metabolism, phenylpropanoid biosynthesis, starch and sucrose metabolism, glycerolipid metabolism, arginine and proline metabolism, glycolysis/gluconeogenesis, and pentose and glucosinolate interconversion pathways (Figure 11A). Salt stress has repeatedly been associated with arginine and proline metabolism, phenylpropane biosynthesis, plant hormone signal transduction, and alpha-linolenic acid metabolism. A total of 140 differential metabolites of *HD96-1* were significantly enriched in starch and sucrose metabolism, stilbenoids, diarylheptanoid and gingerol biosynthesis pathways, arachidonic acid metabolism, linoleic acid metabolism, and oxidative phosphorylation (Figure 11B). *IR29* and *HD96-1* were significantly enriched in starch and sucrose metabolism, stilbenoids, and diarylheptanoid and gingerol biosynthesis pathways (Table S6).

#### Key Biological Pathways Involved in Transcriptomic and Metabolomic Changes

To better understand the important biological pathways of Pro-Ca spraying to improve salt tolerance in rice, we focused on the linkages between differentially expressed genes and changes in differential metabolites after Pro-Ca spraying compared to salt stress treatments under salt stress in rice.

**Figure 12:**
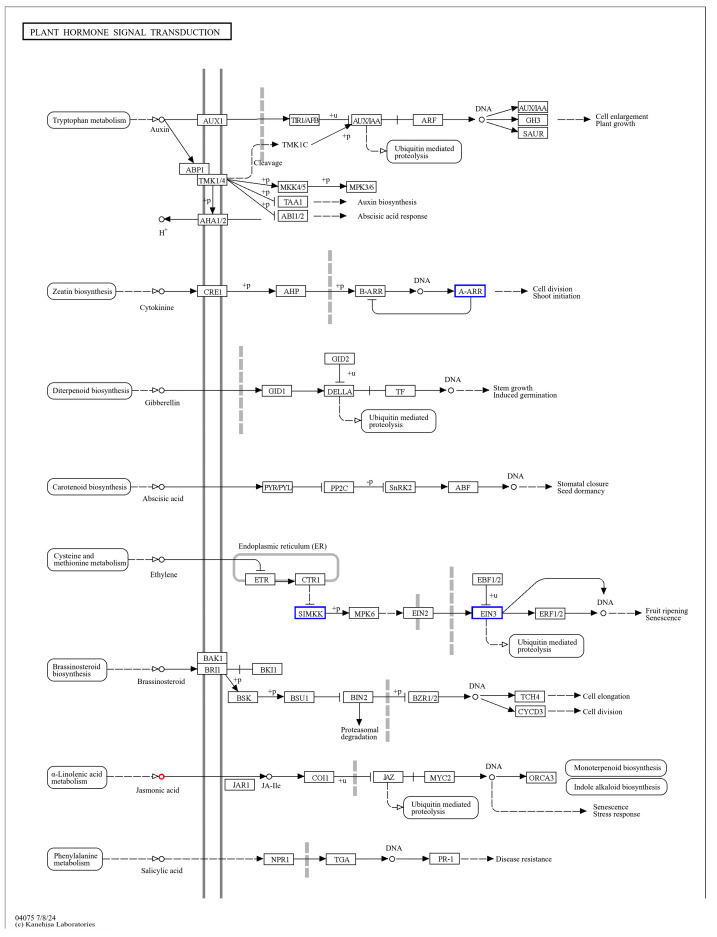
Transcription, metabolome co-enrichment of plant hormone signal transduction pathways.

**Figure 13:**
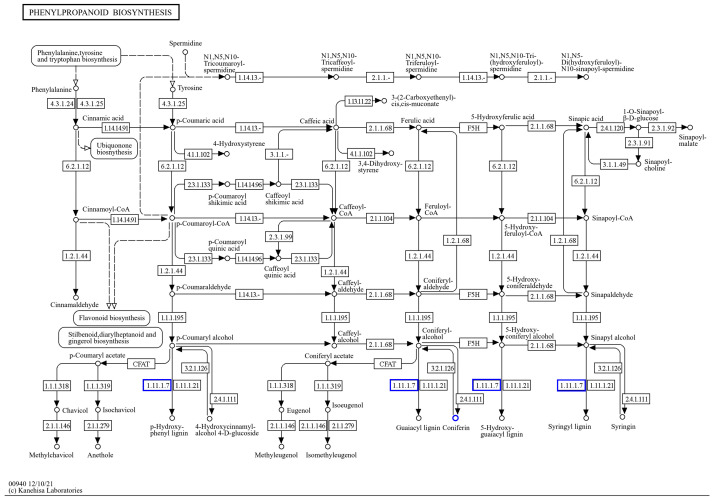
Transcriptional, metabolomic co-enrichment of the phenylpropane biosynthetic pathway.

By the KEGG enrichment of differentially expressed genes, as well as differential metabolites, in *IR29* (ISPCM/IS), it was found that the differential genes and differential metabolites were significantly enriched in plant hormone signal transduction (Figure 12) and the phenylpropanoid biosynthetic pathway (Figure 13).

**Figure 14:**
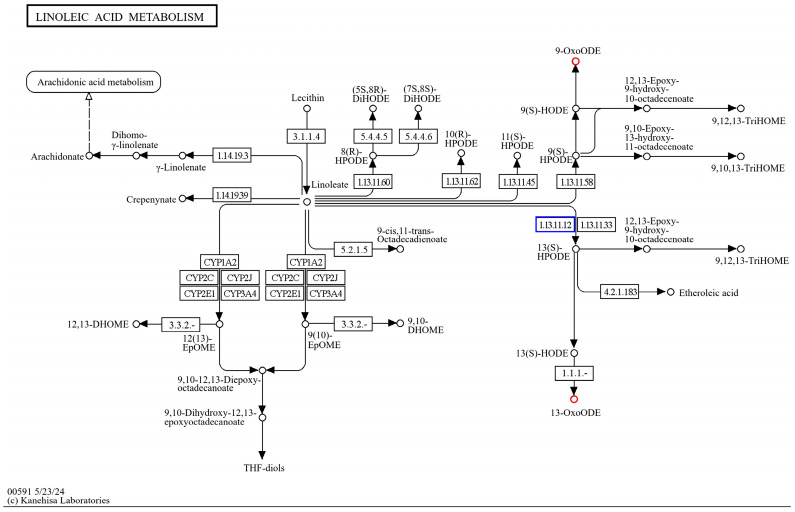
Transcriptional, metabolomic co-enrichment of linoleic acid metabolic pathways.

The KEGG enrichment of differentially expressed genes, as well as differential metabolites, of *HD96-1* (HSPCM/HS) revealed that the differential genes and differential metabolites were co-enriched in the linoleic acid metabolism pathway (ko00591) (Figure 14). The genes related to the control of linoleic acid 13S-lipoxygenase on this pathway showed a significant up-regulation. Both oxylipins, 13-KODE and 9-KODE, whose synthesis is catalyzed by lipoxygenase, showed a significant up-regulation.

## 3. Discussion

Previous studies have shown that Pro-Ca has a mitigating effect on rice salt stress [18], but its molecular mechanism has not been elucidated. In this study, through the transcriptome, metabolome, morphology, and physiological association, we gained insights into the various changes behind the salt stress in rice and explored the mitigating effect of Pro-Ca on rice salt stress.

### 3.1. Pro-Ca Alleviates the Effects of Salt Stress on Rice by Regulating Morphological Traits

Previous studies illustrated that salt stress inhibited morphological traits such as seedlings height, stem diameter, and the root development [29]. Our results also showed that salt stress significantly suppressed the plant height, stem base width, leaf area, dry matter accumulation, and root growth of *IR29* and *HD96-1*, which was consistent with previous studies [30]. Compared with *HD96-1*, salt stress significantly inhibited the *IR29* variety at the 2.5 and 4.5 leaf stages. Pro-Ca significantly increased stem thickness, leaf area, and dry matter accumulation in both varieties, *IR29* and *HD96-1*. Salt stress significantly inhibited the root growth of *IR29* and *HD96-1* at both the 2.5 and 4.5 leaf stages. Pro-Ca treatment significantly increased the total root length, total root surface area, and total root volume of *IR29* and *HD96-1* at both the 2.5 and 4.5 leaf stages. The application of Pro-Ca significantly improved the dry matter accumulation of roots in both varieties. This also indicates that salt stress severely restricts the growth and development of rice. In addition, the inhibitory effect of salt stress on *IR29* was significantly stronger than that of *HD96-1*. The application of Pro-Ca effectively mitigated the salt stress of both varieties, and between the varieties, *HD96-1* performed well.

### 3.2. Pro-Ca Alleviates the Effects of Salt Stress on Rice by Regulating Ion Content

“*LOC4343997*” is an E3 ubiquitin ligase-associated control gene, which was significantly up-regulated in the ISPCM/IS and HSPCM/HS groups. The E3 ubiquitin ligase, as the last enzyme in the first phase of the ubiquitin proteasome pathway, plays a specific recognition role in the process of recognizing the target substrate [31]. Studies have shown that E3 ubiquitin ligase can be involved in abiotic stress responses by directly or indirectly regulating the Na^+^/K^+^ content scavenging system [32]. Under salt stress, *IR29* and *HD96-1* seedlings contained a lot of Na^+^, which stopped other ions from entering. The accumulation of excess Na^+^ is ionotoxic to rice. It further disrupts the osmotic pressure and water potential of rice cell membranes, which ultimately destroys the integrity of cell membranes [9,10]. These primary stresses lead to oxidative stress and may result in a series of secondary stresses that can have an impact on rice growth and development [11]. And K^+^ participates in a myriad of physiological functions in plants, and since high external Na^+^ often competitively inhibits its uptake, K^+^ deficiency becomes severe under salt stress [33]. The imbalance of intracellular and extracellular Na^+^/K^+^ ions will stop the rice from growing and may even be harmful to it [12]. Pro-Ca regulates the above-ground and root Na^+^ and K^+^ content through up-regulation of the genes related to E3 ubiquitin ligase, maintains the Na^+^/K^+^ content balance, and reduces the ion toxicity caused by salt stress.

### 3.3. Effect of Pro-Ca on Photosynthesis in Rice under Salt Stress

In this experiment, we evaluated the effects of salt stress and Pro-Ca spraying on photosynthesis by determining the gas exchange parameters (Pn, Gs, Ci, Tr) of leaves. The results showed that all four indexes decreased under salt stress. And all four indexes showed different degrees of increase after spraying Pro-Ca under salt stress, which was a similar phenomenon to a previous study [18]. Stomatal conductance (Gs) was significantly elevated, indicating that the inhibition of CO_2_ diffusion from the environment to the chloroplasts was mitigated by Pro-Ca. The increase in Pn could be attributed to the opening of stomata, which induced an increase in intracellular CO_2_ content. It has been shown that non-stomatal factors also cause a decrease in Pn, including a large accumulation of Na^+^, a decrease in intracellular K^+^ content, a decrease in photosynthetic pigments, damage to the ultrastructure of chloroplasts, and a decrease in the activity of key photosynthetic enzymes [34]. In this study, chlorophyll content decreased and the Na^+^/K^+^ equilibrium was disrupted under salt stress. Chlorophyll content increased and Na^+^/K^+^ equilibrium was maintained after spraying Pro-Ca under salt stress, suggesting that non-stomatal factors influence photosynthetic efficiency. The above results indicated that both rice salt stresses increased photosynthetic efficiency and alleviated the effects of salt stress on rice through stomatal and non-stomatal factors after spraying Pro-Ca under salt stress.

The light energy captured by the photosynthetic apparatus is mainly used for photosynthesis, but a small portion is dissipated in the form of fluorescence and heat. The chlorophyll fluorescence index, F_v_/F_m_, is the maximum photochemical quantum yield of PS II and can be used as a photoinhibition index to reflect the efficiency of light energy conversion in the active center of PS II [35]. In this study, Pro-Ca increased the Fv/Fm of IR29 and HD96-1 and improved the photochemical conversion efficiency of the PS II protein complex in IR29 and HD96-1 compared with salt stress. In higher plants, LHC II is not only an important regulator of light energy capture in photosynthesis but also a primary site for heat dissipation [36]. In the case of excess light energy, LHC II can reversibly switch from the light energy capture state to the dissipation state through feedback regulation, a reversible regulatory mechanism that regulates the light energy capture state and the dissipation state, which is an important mechanism in stressful environments [37]. Compared with salt stress, the expressions of the LHC II-related coding genes *Lhcb1*, *Lhcb2*, *Lhcb3*, *Lhcb5*, and *Lhcb6* in combined treatment with salt and Pro-Ca showed a significantly down-regulated trend, which reduced the size of LHC II. Pro-Ca may switch the light energy capture state to the dissipative state by regulating LHC II and protect the photosynthetic organs from light inhibition, thus improving the photosynthetic efficiency and salt tolerance of *HD96-1*. This also provides a direction for future research. At the same time, Pro-Ca also reduced the photoinhibition of *IR29* under salt stress, but the specific mechanism remains to be further explored. Furthermore, PSII potential photochemical activity (Fv/Fo) is another important photochemical parameter that provides an estimation of leaf photosynthetic capacity [38,39]. The Fv/Fo of both *HD96-1* and *IR29* decreased significantly under salt stress, and the Fv/Fo of *IR29* and *HD96-1* showed different degrees of increase under Pro-Ca. This demonstrated that Pro-Ca was effective in enhancing photosynthesis in rice under salt stress.

In summary, Pro-Ca increased the photosynthetic efficiency of *IR29* under salt stress through stomatal and nonstomatal factors. Among these, Pro-Ca also increased photosynthetic efficiency by down-regulating the LHC II-related coding genes of *HD96-1*, which reduced the size of LHC II, increased heat dissipation, and protected photosynthetic organs from photoinhibition.

### 3.4. Pro-Ca Activates Plant Hormone Signal Transduction to Alleviate the Effects of Salt Stress on Rice

The results suggest that plant hormone signal transduction may be highly correlated with the Pro-Ca regulation of rice acclimation to salt stress, and the important control genes of *A-ARR* on this pathway showed a significant up-regulation trend. *A-ARR* is a cytokinin-responsive gene, which is the target of *B-ARR TFs 12*, and *A-ARR* is a negative cytokinin regulator, and the up-regulation of the expression of *A-ARR* on cytokinin signaling has an inhibitory effect [40]. The reduction in cytokinin level in plants under salt stress has been found to be considered an effective defense mechanism for plants to respond to salt stress in previous studies [41]. By examining the plant hormone content, it was found that the cytokinin content was significantly decreased by spraying Pro-Ca under salt stress compared with salt stress treatment. Cytokinins (CKs) have been long-known inhibitors of root growth and development, and multiple mutants blocking CK biosynthesis show enhanced primary root length compared to wild-type plants [42]. Our results of root scanning showed that total root length, total root surface area, and total root volume decreased significantly in *IR29* and *HD96-1* under salt stress and significantly increased after the application of Pro-Ca treatment. It indicates that Pro-Ca under salt stress inhibits cytokinin synthesis by up-regulating the *A-ARR* gene and suppresses cytokinin signaling, which promotes root growth and ultimately increases the resistance of *IR29* and *HD96-1* to salt stress.

### 3.5. Effect of Pro-Ca on MAPK Signaling Pathway in Rice under Salt Stress

The transcriptome data showed that there were four differentially expressed genes in the combined Pro-Ca and salt stress treatment (ISPCM/IS) of *IR29* that were significantly enriched in the MAPK plant signaling pathway as compared to the salt stress treatment. And the deferentially expressed genes in this pathway were mainly focused on peptides, endogenous hormone levels, antioxidant capacity, and stomatal development, most of which were responsive to salt stress. The MAPK signaling pathway mainly involves plant hormones, hydrogen peroxide (H_2_O_2_), stomata, and other signaling pathways. *EIN* and *EIL* are key genes involved in the ETH signaling pathway. Compared with salt treatment, the *EIN3-5like* associated with ETH synthesis was significantly down-regulated in the Pro-Ca treatment, and a previous study found that the down-regulation of *EIN3* inhibited the ethylene synthesis process [43]. Ethylene (ETH) is one of the major plant hormones. Ethylene has negative effects on the growth of some plants (zucchini, tomato, Arabidopsis, and tobacco, among others) under abiotic stress [44]. Spraying Pro-Ca under salt stress may indirectly inhibit ethylene synthesis by down-regulating *EIN3-5like* expression, thereby improving salt tolerance in rice.

In the peptide and H_2_O_2_ pathway, we found that the transcription factor *WRKY22* was significantly down-regulated under salt stress and up-regulated under Pro-Ca treatment. The *WRKY* gene family has been shown to be associated with salt stress many times. The maize *WRKY114* gene negatively regulates transgenic rice salt tolerance [44], and the *WRKY* transcription factor (*ZmWRKY17*) negatively regulates transgenic Arabidopsis thaliana salt stress tolerance [45]. We can hypothesize that *WRKY22*, as a negative regulator, is involved in the response of IR29 rice to salt stress through the Pro-Ca-mediated peptide, as well as the H_2_O_2_ pathway, but the specific regulatory mechanism is not clear and needs to be further explored.

The MAPK signaling pathway is also involved in the stomatal development process of rice, in which the related control gene *OJ1316_E06.24* of the signaling cascade *MKK4/5* showed a significant down-regulation under the action of Pro-Ca, which was proven to be negatively regulated by previous studies [46]. Spraying Pro-Ca alleviated the damage to stomata caused by salt stress through a significant down-regulation of the relevant control genes of *MKK4/5*, thus improving the salt tolerance of *HD96-1* rice.

### 3.6. Effect of Pro-Ca on Carotenoid Biosynthesis in Rice under Salt Stress

Based on the ISPCM/IS transcriptome KEGG enrichment results, the radishin biosynthetic pathway was significantly enriched. The catabolic process of abscisic acid is mainly accomplished by ABA 8′-hydroxylase, which plays an important role in the catabolism of abscisic acid and is a member of the *CYP707A* subfamily [47]. It catalyzes the C8′-hydroxylation of ABA to form 8′-hydroxyABA, which then spontaneously isomerizes to the more stable phase acid (PA) [48], which has a much lower hormonal activity than ABA [47]. Transcriptome data showed that both *CYP707A7* and *CYP707A5*, the related control genes of ABA 8′-hydroxylase in *IR29* rice, showed a significant down-regulation after spraying Pro-Ca under salt stress compared with the salt stress treatment. And we identified that Pro-Ca, by down-regulating the related control genes of ABA 8′-hydroxylase, caused the activity of ABA 8′-hydroxylase to be inhibited, thus inhibiting the catabolic process of ABA and maintaining the ABA content, to improve the resistance of *IR29* to salt stress.

### 3.7. Effect of Pro-Ca on Linoleic Acid Metabolism of Rice under Salt Stress in Rice

HSPCM/HS differential genes and differential metabolites are co-enriched in the linoleic acid metabolism (ko00591) pathway. The genes related to the control of linoleic acid 13S-lipoxygenase on this pathway showed a significant up-regulation. Lipoxygenase (linoleate: oxygen oxidoreductase, EC 1.13.11.12) is a type of non-heme iron-containing lipid dioxygenase that catalyzes the degradation of unsaturated fatty acids and is an enzyme with a key role in the LOX pathway of fatty acid metabolism [49]. Lipoxygenases (LOXs) are non-heme iron-containing dioxygenases that catalyze the oxidation of polyunsaturated fatty acids and lipids, to trigger the formation of a group of biologically active compounds known as oxylipins [50]. Oxylipin is an oxygenated lipid derived from polyunsaturated fatty acids, which plays an important role in mitigating biotic and abiotic stress [51]. By analyzing the differential metabolites significantly enriched in this pathway, it was found that the content of both oxylipins, 13-KODE and 9-KODE, showed a significant up-regulation. In this study, it was shown that spraying Pro-Ca may alleviate rice salt stress by up-regulating the relevant control genes of lipoxygenase, so that the content of both oxylipin metabolites, 13-KODE and 9-KODE, showed a trend of significant increase.

## 4. Conclusions

**Figure 15:**
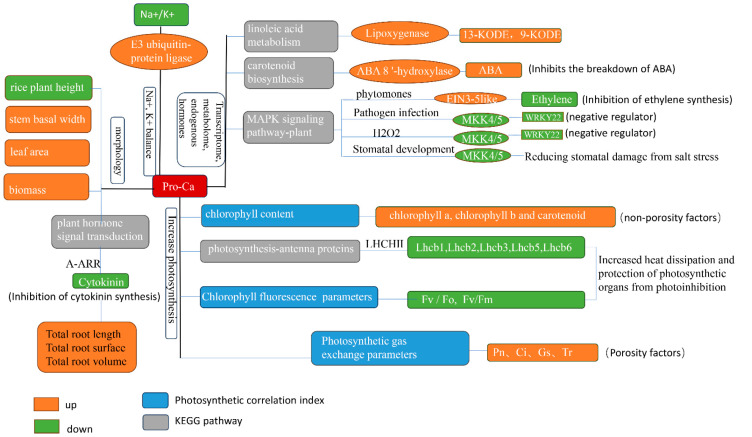
Mechanism of salt stress alleviation in rice by Pro-Ca.

Our study used morpho-physiological, transcriptomic, and metabolomic approaches to investigate the specific mechanisms of salt-tolerant rice (*HD96-1*) and salt-sensitive rice (*IR29*) varieties’ resistance to salt stress under the application of Pro-Ca. The results indicated that Pro-Ca could alleviate the effects of salt stress in both rice varieties by regulating morphogenesis, maintaining sodium and potassium ion balance, increasing chlorophyll content, improving photosynthetic efficiency, inhibiting endogenous cytokinin synthesis, and promoting root growth (Figure 15). However, the specific molecular mechanisms are somewhat different. Pro-Ca also inhibited cytokinin synthesis and signaling by increasing *A-ARR* in *HD96-1* and *IR29*, which later promoted root morphology. Pro-Ca increased photosynthetic efficiency by decreasing LHC II in *HD96-1* and also increased the content of 13-KODE and 9-KODE by up-regulating the lipoxygenase-related control genes of *HD96-1*, thus alleviating salt stress. Pro-Ca inhibited the catabolic process of ABA by down-regulating the genes related to the control of ABA 8 ‘-hydroxylase in IR29 and maintained ABA content, in order to increase the resistance of *IR29* to salt stress. Pro-Ca also alleviated the salt stress of *IR29* through peptides, endogenous hormone levels, antioxidant capacity, and stomatal development. Pro-Ca has some common mechanisms for alleviating salt stress in different salt-tolerant rice varieties. Some differences might originate from the differences between the salt-sensitive variety (*IR29*) and the salt-tolerant variety (*HD96-1*). This study will provide a framework and valuable empirical data for further studies on the relationship between Pro-Ca treatment and salt stress in plants.

## 5. Materials and Methods

### 5.1. Experimental Materials

The salt-tolerant rice *IR29* (salt-sensitive) and salt-sensitive rice *HD96-1* (salt-tolerant) belonging to Oryza sativa L. subsp. Indica were used in this study. Germplasm resources were provided by the College of Coastal Agriculture, Guangdong Ocean University. The plant growth regulator for testing was prohexadione calcium (Pro-Ca), and the stock solution (5% Pro-Ca) was provided by Sichuan Runer Technology Co. (Chengdu, Sichuan, China).

### 5.2. Plant Materials and Growth Conditions

The experiment was carried out in 2022 in the sunlight greenhouse (day/night temperature of 26/22 °C, day/night photoperiod of 10/14 h, and relative humidity of 70%) of the College of Coastal Agricultural Sciences of Guangdong Ocean University, Zhanjiang, Guangdong Province. The experiment was set up with two types of rice and three treatments: control (CK), NaCl stress treatment (S), and NaCl stress + Pro-Ca treatment (SPCM).

Seeds were selected to be full and whole, and then disinfected with 3% hydrogen peroxide for 15 min, rinsed with distilled water several times until thoroughly rinsed, soaked with distilled water, and germinated for 48 h under dark conditions at 30 °C. Subsequently, 65 uniform seeds were sown in a plastic pot (with an upper diameter of 19 cm, lower diameter of 14 cm, height of 17 cm, and holes in the bottom); each pot was filled with about 2 kg of test soil (the volume ratio of latosol to sand was 3:1). A total 1.46 g of nitrogen fertilizer (urea), 2 g of phosphorus fertilizer (superphosphate), and 1.25 g of potassium fertilizer (potassium chloride) was used in each plastic basin, respectively. When the rice seedlings had naturally grown to the 1.5 leaf stage, the seedlings were foliar sprayed with 100 mg/L Pro-Ca, and the Pro-Ca concentration was determined by a previous concentration screening test [52]. A 50 mmol/L NaCl solution was selected to simulate salt stress, and clear water was used as the control. The pots were watered every 3 days with the corresponding concentration of salt solution or clear water at 0.6 L. The soil salinity was monitored by a Shunktar soil tester (TR-6D).

### 5.3. Measurement of Growth Index

Samples were taken at the 2.5-leafed and 4.5-leafed stages, with three replicates per treatment. The plant height and stem diameter of each individual were measured with a ruler and vernier. The leaf area was scanned with a Yaxm-1241 leaf area meter (Beijing Yaxin RIYI Technology Co., Ltd., Beijing, China). The rice seedlings were subsequently dried at 105 °C for 30 min and dried at 80 °C to a constant weight, and the above-ground dry weight and root dry weight were determined by an electronic balance. The rice seedling root systems were scanned with a root system scanner (Epson Perfection V800 Photo (Epson Indonesia Inc., Suwa, Nagano, Japan)), and the images were analyzed and calculated using WinRHIZO root analysis system (Regent Instruments, Québec City, QC, Canada) to obtain the total root length, total root surface, and total root volume.

### 5.4. Measurement of Photosynthetic Gas Exchange Parameters

We measured the net photosynthetic rate (Pn), intercellular carbon dioxide concentration (Ci), transpiration rate (Tr), and stomatal conductance (Gs) using the LI-6800 portable photosynthesis measurement system (LI-6800, LI-COR, Lincoln, NE, USA) from 9:00 to 11:00 a.m. on a sunny day, with three replicates for each treatment [53]. The conditions in the leaf chamber were a photosynthetically active radiation (PAR) of 1000 μmol·m^−2^·s^−1^, a CO_2_ concentration of 400 μmol·mol^−1^, leaf temperature of 30.0 °C, relative air humidity between 70 and 80%, and air velocity of 500 μmol·s^−1^.

### 5.5. Measurement of Chlorophyll Fluorescence Parameters

Chlorophyll fluorescence parameters were determined at the 2.5-leaf stage and 4.5-leaf stage using the Chlorophyll Fluorometer System (OS5P, OPTI-Sciences, Boston, MA, USA). Before determining the chlorophyll fluorescence parameters, rice plants from each treatment were dark-adapted under dark conditions for 30 min, then the minimum fluorescence yield (Fo) and the maximal fluorescence yield (Fm) were measured. The variable fluorescence (Fv), the maximum quantum yield of PSII photochemistry (Fv/Fm), and PSII potential photochemical activity (Fv/Fo) were calculated according to Maxwell and Johnson [39,54,55].

### 5.6. Measurement of the Photosynthetic Pigments

At the 2.5 and 4.5 leaf stages, chlorophyll a (Chl a), chlorophyll b (Chl b), carotenoids (Car), and total chlorophyll (Chl a + b) were determined using the method proposed by Kolomeichuk [56]. A fresh leaf (0.1 g) was soaked in 10 mL 95% ethanol in the dark for 24 h. The concentrations of Chl a, Chl b, and Car were measured spectrophotometrically at 665, 649, and 470 nm, respectively.

Chlorophyll a (Chl a) = 13.95D665 − 6.88D649

Chlorophyll b (Chl b) = 24.96D649 − 7.32D665

Total chlorophyll content = Chl a + Chl b

Carotenoid (Car) = (1000 D 470 − 2.05 Chl a − 111.48 Chl b)/245

### 5.7. Measurement of Ion-Related Indicators

Na^+^ and K^+^ content was measured during the 2.5 leaf stage of the rice. The rice shoots were washed with clean water and then dried at 75 °C until attaining a constant weight. The samples were ground into uniform powder using a tissue grinder. A 0.2 g sample was weighed and subjected to microwave digestion, ashing, and other pretreatments to prepare the liquid to be tested. Na^+^ and K^+^ content was determined using an inductively coupled plasma optical emission spectrometer (ICP-OES, Thermo Scientific ICAP 6000 Series, Thermo Fisher Scientific, Waltham, MA, USA).

### 5.8. Endogenous Hormone Extraction and Determination

A total of 50 mg of the ground sample was weighed, and 10 μL of the internal standard mixing solution at a concentration of 100 mg/mL and 1 mL of methanol/water/formic acid (15:4:1, *v*/*v*/*v*) extractant was added and mixed well; the samples were vortexed for 10 min, and then centrifuged for 5 min at 4 °C, 12,000 r/min. And the supernatant was concentrated in a new centrifuge tube. After concentration, the extract was reconstituted with 100 μL of 80% methanol/water solution, passed through a 0.22 μm filter membrane, and put into the injection bottle for LC-MS/MS analysis. Data acquisition was performed using ultra-performance Performance Liquid Chromatography (UPLC) [57]. A MWDB (Metware Database v5.0) database was constructed based on the standards to qualitatively analyze the data detected by mass spectrometry. The quantification was accomplished by analysis using the Multiple Reaction Monitoring (MRM) mode of triple quadrupole mass spectrometry. Analyst 1.6.3 software was used to process the mass spectrometry data. There were three biological replicates for each treatment.

### 5.9. Total RNA Isolation and Transcriptome Analysis

The authors took the penultimate complete leaf of each treatment at the 1.5-leaf stage, with three biological replicates for each treatment. In this experiment, RNA was extracted from rice leaves by ethanol precipitation using CTAB-PBIOZOL reagent. Total RNA was analyzed qualitatively and quantitatively using a Drop and Agilent 2100 Bioanalyzer (Thermo Fisher Scientific, Waltham, MA, USA) [58]. Clean reads were obtained using SOAPnuke (v1.5.2) [59]. The clean reads were then compared to the reference gene sequences using Bowtie2 (v2.2.5) to obtain the comparison results. Differentially expressed genes with |Log2FC ≥ 1|, *p* < 0.05 were screened using Phyper (https://en.wikipedia.org/wiki/Hypergeometric_distribution, accessed on 30 May 2024) and analyzed by KEGG (https://www.kegg.jp/, accessed on 30 May 2024).

### 5.10. Real-Time Fluorescence Quantitative PCR (qRT-PCR) Validation

In order to verify the reliability of the RNA sequencing data, a real-time fluorescence quantitative polymerase chain reaction (RT-qPCR) was used for the expression of seven randomly selected differentially expressed genes in the transcriptome and the internal reference gene UBQ5. There were three biological replicates for each gene.

### 5.11. Metabolite Extraction and Metabolomic Analysis

The same materials were used for metabolite analysis and the transcriptome. Three replicates were processed for each sample. Each sample was placed in an Eppendorf tube after grinding at 50 Hz for 5 min with a tissue grinder (JXFSTPRP) and ultrasonicated in a water bath at 4 °C for 30 min, followed by refrigeration at −20 °C for one hour. The samples were then centrifuged for 15 min (14,000 RPM Centrifuge (5430)), using a 0.22 μm membrane filter following LC-MS analysis. In this project, the LC-MS/MS technology was used for non-targeted metabolomics analysis, and the raw mass spectrometry data (raw files) were collected using LC-MS/MS and imported into Compound Discoverer 3.1 (Thermo Fisher Scientific, USA) for data processing, as well as the Metabolome Information Analysis Process for data preprocessing, statistical analysis, metabolite classification annotation, and functional annotation. There were three biological replicates for each treatment.

### 5.12. Statistical Analysis

Means and standard deviations were calculated using IBM SPSS Statistics 26, and a one-way analysis of variance (ANOVA) was performed. Duncan’s multiple-range test was subsequently performed. *p* < 0.05 was considered significant. Figures were compiled in Excel 2021, and graphs were plotted in Origin 2021. The Pearson’s correlation coefficient was calculated for each sample using XLSTAT 2019 (Addisonsoft XLSTAT, Paris, France). Principal component analysis (PCA) was performed using the Factoextra 1.0.7 and FactoMineR 2.9 in R 4.2.0.

## Figures and Tables

**Table 1:** Effect of Pro-Ca on Na^+^ content, K^+^ content, and Na^+^/K^+^ of rice seedlings under salt stress. Mean ± SE of three replicates. CK: 0 mM NaCl + 0 mg·L^−1^Pro-Ca, S: 50 mM NaCl + 0 mg·L^−1^Pro-Ca, SPCM: 50 mM NaCl + 100 mg·L^−1^Pro-Ca. Values are the mean ± SE of three replicate samples. Different letters in the data column indicate significant differences (*p* < 0.05) according to Duncan’s test.

Variety	Treatment	Leaf Na^+^	Leaf K^+^	Leaf Na^+^/K^+^	Root Na^+^	Root K^+^	Root Na^+^/K^+^
IR29	CK	0.83 ± 0.00 c	20.93 ± 0.11 b	0.04 ± 0.00 c	1.59 ± 0.00 b	16.61 ± 0.00 b	0.10 ± 0.00 b
	S	11.48 ± 0.03 a	20.35 ± 0.00 c	0.56 ± 0.00 a	7.94 ± 0.01 a	14.23 ± 0.00 c	0.56 ± 0.00 a
	SPCM	1.57 ± 0.00 b	21.86 ± 0.11 a	0.07 ± 0.00 b	1.48 ± 0.00 c	18.70 ± 0.11 a	0.08 ± 0.00 c
HD96-1	CK	0.85 ± 0.00 c	29.32 ± 0.11 b	0.03 ± 0.00 b	1.60 ± 0.00 b	16.62 ± 0.00 b	0.10 ± 0.00 b
	S	10.38 ± 0.02 a	24.83 ± 0.12 c	0.42 ± 0.00 a	6.43 ± 0.00 a	14.10 ± 0.00 c	0.46 ± 0.00 a
	SPCM	0.90 ± 0.00 b	29.11 ± 0.11 a	0.03 ± 0.00 b	1.84 ± 0.01 c	17.13 ± 0.00 a	0.11 ± 0.00 c

**Table 2:** Effect of Pro-Ca on chlorophyll a (Chl a), chlorophyll b (Chl b), carotenoids (Car), and total chlorophyll (Chl a+b) content of rice under salt stress. CK: 0 mM NaCl + 0 mg·L^−1^Pro-Ca, S: 50 mM NaCl + 0 mg·L^−1^Pro-Ca, SPCM: 50 mM NaCl + 100 mg·L^−1^Pro-Ca. Values are the mean ± SE of three replicate samples. Different letters in the data column indicate significant differences (*p* < 0.05) according to Duncan’s test.

Sampling Period	Treatment	Chl a (mg·g^−1^)	Chl b (mg·g^−1^)	Chl a+b (mg·g^−1^)	Car (mg·g^−1^)
2.5 leaf	ICK	23.960 ± 1.070 a	8.516 ± 0.574 a	32.472 ± 1.645 a	51.145 ± 2.188 a
	IS	18.602 ± 0.600 b	6.266 ± 0.273 b	24.867 ± 0.871 b	40.445 ± 1.154 b
	ISPCM	19.418 ± 0.434 b	6.506 ± 0.160 b	25.924 ± 0.594 b	44.676 ± 0.634 b
	HCK	16.874 ± 0.288 a	5.578 ± 0.106 a	22.452 ± 0.394 a	35.509 ± 0.562 a
	HS	15.569 ± 0.906 a	5.117 ± 0.315 a	20.686 ± 1.220 a	33.332 ± 1.905 a
	HSPCM	16.487 ± 1.380 a	5.497 ± 0.478 a	21.983 ± 18.858 a	33.921 ± 2.721 a
4.5 leaf	ICK	23.102 ± 0.358 a	7.989 ± 0.136 a	31.091 ± 0.493 a	59.904 ± 0.844 a
	IS	18.853 ± 1.408 b	6.177 ± 0.516 b	25.029 ± 1.924 b	48.741 ± 4.045 b
	ISPCM	23.249 ± 0.751 a	8.453 ± 0.345 a	31.702 ± 1.924 b	60.319 ± 1.969 a
	HCK	29.979 ± 0.152 a	14.166 ± 0.360 a	44.145 ± 0.489 a	65.902 ± 0.199 a
	HS	24.074 ± 0.575 c	8.518 ± 0.298 c	32.592 ± 0.873 c	59.974 ± 1.195 b
	HSPCM	27.090 ± 0.531 b	11.284 ± 0.496 b	38.374 ± 1.027 b	61.815 ± 0.784 b

**Table 3:** Compared to salt treatment, the combined treatments of Pro-Ca and salt stress changes endogenous plant hormone content in IR29 and HD96-1. CK: 0 mM NaCl + 0 mg·L^−1^Pro-Ca, S: 50 mM NaCl + 0 mg·L^−1^Pro-Ca, SPCM: 50 mM NaCl + 100 mg·L^−1^Pro-Ca.

Variety	Index	Compounds	Class	Material Category	Pvalue	Log2FC	Type
IR29	mT9G	meta-Topolin-9-glucoside	CK	cytokinin	0.191	−Inf	down
	DHZROG	Dihydrozeatin-O-glucoside riboside	CK	cytokinin	0.074	−2.011	down
	BAP	6-Benzyladenine	CK	cytokinin	0.015	−Inf	down
HD96-1	cZ	cis-Zeatin	CK	cytokinin	1.403	−1.140	down
	K9G	Kinetin-9-glucoside	CK	cytokinin	0.007	−Inf	down

## Data Availability

The transcriptome and metabolome data presented in this study can be found in online repositories. The raw sequence data have been deposited in the Genome Sequence Archive in the National Genomics Data Center, Beijing Institute of Genomics, Chinese Academy of Sciences, under accession number CRA016731, publicly accessible at https://bigd.big.ac.cn/gsa (accessed on 30 May 2024). The metabolite data have been deposited in the National Genomics Data Center, Beijing Institute of Genomics, Chinese Academy of Sciences, under accession number OMIX006554, publicly accessible at: https://bigd.big.ac.cn/omix (accessed on 30 May 2024).

## Supplementary Materials

The following supporting information can be downloaded at: https://www.mdpi.com/article/10.3390/ijms25169124/s1.
